# Low-Complexity Automorphism Ensemble Decoding of Reed-Muller Codes Using Path Pruning

**DOI:** 10.3390/e27080808

**Published:** 2025-07-28

**Authors:** Kairui Tian, Rongke Liu, Zheng Lu

**Affiliations:** 1 School of Electronic and Information Engineering, Beihang University, Beijing 100191, China; philtian@buaa.edu.cn (K.T.); htluzheng@buaa.edu.cn (Z.L.); 2 Shenzhen Institute, Beihang University, Shenzhen 518063, China

**Keywords:** Reed–Muller (RM) codes, Plotkin construction, automorphism ensemble decoding, complexity reduction

## Abstract

The newly developed automorphism ensemble decoder (AED) leverages the rich automorphisms of Reed–Muller (RM) codes to achieve near maximum likelihood (ML) performance at short code lengths. However, the performance gain of AED comes at the cost of high complexity, as the ensemble size required for near ML decoding grows exponentially with the code length. In this work, we address this complexity issue by focusing on the factor graph permutation group (FGPG), a subgroup of the full automorphism group of RM codes, to generate permutations for AED. We propose a uniform partitioning of FGPG based on the affine bijection permutation matrices of automorphisms, where each subgroup of FGPG exhibits permutation invariance (PI) in a Plotkin construction-based information set partitioning for RM codes. Furthermore, from the perspective of polar codes, we exploit the PI property to prove a subcode estimate convergence (SEC) phenomenon in the AED that utilizes successive cancellation (SC) or SC list (SCL) constituent decoders. Observing that strong SEC correlates with low noise levels, where the full decoding capacity of AED is often unnecessary, we perform path pruning to reduce the decoding complexity without compromising the performance. Our proposed SEC-aided path pruning allows only a subset of constituent decoders to continue decoding when the intensity of SEC exceeds a preset threshold during decoding. Numerical results demonstrate that, for the FGPG-based AED of various short RM codes, the proposed SEC-aided path pruning technique incurs negligible performance degradation, while achieving a complexity reduction of up to 67.6%.

## 1. Introduction

In recent years, the rapid development of Internet of Things (IoT) applications has highlighted the demand for efficient short-length coding schemes [1]. This has led to a resurgence of interest in Reed–Muller (RM) codes [2], a class of error-correcting codes with a rich history in both academia and industry. One of the key reasons for this renewed attention is the close relationship between RM codes and polar codes [3]. Similar to polar codes, RM codes have been proven to achieve capacity over various channels, such as the binary erasure channel [4], the binary symmetric channel [5], and the binary memoryless symmetric channel [6]. Additionally, of practical significance, RM codes have excellent maximum likelihood (ML) decoding performance due to large minimum distance, which also holds in the short-length regime [7,8]. This makes them highly competitive in IoT scenarios demanding high reliability with short packages, such as the emerging hyper-reliable low-latency communication (HRLLC) [9]. However, while first-order RM codes benefit from an efficient ML decoder, which is the fast Hadamard transform (FHT) decoder [10], ML decoding is generally infeasible due to prohibitively high complexity. This challenge has spurred significant interest in the development of efficient decoding algorithms for RM codes, with the goal of approaching the ML decoding performance with tractable complexity.

The first RM decoder is the majority-vote decoder invented by Reed [11], which can correct errors with a weight less than half of the minimum distance. Since then, several additional decoding algorithms have been developed to improve the decoding performance [12,13,14,15]. More recently, the code symmetry of RM codes has been exploited to achieve near ML performance via belief propagation (BP) decoding on highly-redundant parity check (PC) matrices [16]. These PC matrices, composed of minimum-weight dual codewords, are customized for decoding each corrupted codeword. However, this presents a significant challenge for hardware implementation as it requires support for dynamic PC matrices. Additionally, a recursive projection-aggregation (RPA) decoder, which takes advantage of the polynomial representation of RM codes, was proposed in [17]. The RPA decoder performs close to ML decoding under various code rates and lengths, while naturally benefitting from parallel implementation. However, the complexity of RPA decoding significantly increases with the order of RM codes, making it generally impractical for RM codes with large orders [18]. Given that RM codes share a similar construction with polar codes, successive cancellation (SC) decoder and SC list (SCL) decoder [19], which were initially proposed for polar codes, are also applicable to RM codes. Notably, the SCL decoder can achieve near ML performance for short RM codes with a reasonable list size. However, the SCL decoder requires complex path management that cannot be readily parallelized, posing challenges for low-latency decoding even with a relatively small list size [20].

Most recently, an automorphism ensemble decoder (AED) was developed for RM codes, leveraging the large automorphism group of RM codes for near ML performance [21]. Specifically, the channel output is interleaved by a list of permutations randomly sampled from the automorphism group [22], generating multiple permuted instances of the corrupted codeword. Each permuted channel output is decoded by a constituent decoder, which can be any RM or polar decoder. Based on a predefined metric, the most likely codeword estimate is selected. Through observing multiple decoding outputs, the AED acquires diversity gain for enhanced decoding performance [23]. When the SC constituent decoder is employed, the AED demonstrates performance that is comparable to, or even surpasses, that of the SCL decoder under the same list size. More practically important, the SC constituent decoders within the AED operate independently without using path sorting. Thus, in comparison with the SCL decoder, the AED features lower overall complexity and decoding latency, making it highly attractive for hardware implementations [24].

It is worth noting that, similar to the list size of the SCL decoder, the ensemble size of AED for achieving near ML performance grows exponentially with the code length [25]. Therefore, the AED still faces a complexity issue due to the employment of a list of constituent decoders. To reduce the complexity of AED, the SC or SCL constituent decoders can be effectively simplified by incorporating node-based fast decoding techniques [26,27,28,29,30], thereby reducing the overall complexity. However, simplifying each constituent decoder does not take advantage of any characteristics of the AED framework itself. To this end, the authors in [31] propose two early termination (ET) techniques, namely branch and bounds (BB) and repetition handling (RH), which require a sequential launching of the SC constituent decoders. Utilizing the path metric (PM) [32] for evaluating each SC decoding output, the BB method leverages the monotonicity of PM to terminate an SC constituent decoder when its partial PM is already inferior to the currently best PM. The RH method, which exploits the channel-related codeword estimate diversity in the AED, outputs the currently best codeword estimate if it has been generated by multiple SC constituent decoders. Despite the effectiveness, the sequential implementation of AED incurs an undesirable varying latency and needs to be timed according to the worst-case latency in practice [33]. In [31], the authors also develop a PM threshold (PMT)-based ET technique for parallel AED implementation, which terminates an SC constituent decoder once its partial PM exceeds a preset threshold. Nonetheless, this PMT-based ET method fails to exploit collective features among the constituent decoders. As a result, it achieves limited complexity reduction and becomes less effective as the noise level decreases.

In this work, we propose a novel subcode estimate convergence (SEC)-aided path pruning technique for the AED of RM codes, adapting the decoding complexity to the channel condition without compromising the near ML performance. The SEC-aided path pruning relies on observing relative characteristics among constituent decoders, which is applicable to fully parallel and partially parallel AED implementations. The simulation results demonstrate significant complexity reductions for decoding various short RM codes. The major contributions of this paper are summarized as follows.

- From the perspective of polar codes, we investigate information set partitioning of RM codes based on the Plotkin construction. Leveraging a uniform partitioning of the factor graph permutation group (FGPG), which is a subgroup of the full automorphism group of RM codes, we prove that the subcode-based information set partitioning exhibits permutation invariance (PI) between any two automorphisms from the same subgroup of the FGPG. Additionally, we demonstrate that, for the AED of RM codes that uses automorphisms only from the FGPG, the PI property makes it possible for the SC or SCL constituent decoders to generate identical partial information bit estimates, resulting in a notable subcode estimate convergence (SEC) phenomenon during decoding.
- We propose a subcode-based partial constituent metric (SPCM) to detect the SEC in the AED that employs the SC or SCL constituent decoders. We prove that under both theoretical and implementation-friendly forms, the SPCMs of SC (or SCL) constituent decoders that use automorphisms from the same subgroup of the FGPG are identical once SEC occurs. Furthermore, we find that the intensity of SEC can serve as a runtime noise level indicator for the corrupted codeword, where a strong SEC typically indicates a high redundancy of the decoding capacity in the AED. Based on this observation, we develop an SEC-aided path pruning method for AED, which allows only a few constituent decoders to continue decoding when the intensity of SEC exceeds a preset threshold.
- The block error rate (BLER) and decoding complexity of the proposed SEC-aided AED (SEC-AED) are extensively evaluated across multiple short RM codes, covering different code lengths and rates. The numerical results demonstrate that the proposed SEC-AED incurs negligible BLER degradation to the AED, maintaining near ML performance for RM decoding. Additionally, at a low target BLER of around $10^{-5}$, our proposed SEC-AED achieves complexity reductions of up to 43.5% and 67.6% under fully parallel and partially parallel implementations, respectively. The SEC-aided path pruning technique can serve as an efficient power-reduction technology in the hardware-based low-latency AED for RM codes.

The remainder of the paper is structured as follows. Section 2 gives an introduction to RM codes, SC and SCL decoders, and AED and defines the concept of a partial constituent metric (PCM). In Section 3, we develop the proposed SEC-aided path pruning technique and detail its algorithmic implementation. Section 4 provides the simulation results, comparisons with existing work, and explores the practical aspects and potential application of our approach. Finally, the paper concludes with Section 5.

## 2. Preliminaries

### 2.1. Notations

Throughout this paper, boldface letters indicate vectors. The *i*-th element of a vector x is denoted by $x_{i}$. For a vector x and an index vector $V=[v_{0},v_{1},\ldots ]$, $x_{V}$ denotes the sub-vector $\left[x_{v_{0}},x_{v_{0}},\ldots \right]$. If S is a countable set, |S| denotes its cardinality. For a positive integer Z, [Z]≜{0,1,…,Z−1}.

### 2.2. Definition of RM Codes via Hadamard Transforms

An RM code, denoted by RM(m,r), is a linear block code specified by two integers *m* and *r* with 0≤r≤m, where *r* is the order of the code. RM(m,r) has length $N=2^{m}$, dimension K=∑i=0rmi, rate R=K/N and minimum distance $d=2^{m-r}$. The 2×2 Hadamard transform (HT) over the binary field $F_{2}$, defined by the 2×2 matrix

(1) $$H_{2}=\left[\begin{array}{cc} 1 & 0\\ 1 & 1 \end{array}\right],$$

serves as the basic building block of RM codes. The $2^{m}\times 2^{m}$ HT is derived from the *m*-th Kronecker power of $H_{2}$, i.e., $H_{2^{m}}={\left(H_{2}\right)}^{⊗m}$. The Hamming weights of the rows of $H_{2^{m}}$ are powers of 2, and the number of rows of weight $2^{t}$ is the combinatorial coefficient mt. RM(m,r) is defined as the code generated by the rows of $H_{2^{m}}$ of weight at least *d*.

The HT-based definition of RM codes shows the similarity between RM codes and polar codes. Let $w_{i}$ denote the weight of the *i*-th row of $H_{2^{m}}$, where 0≤i<N. In the polar coding context, *i* belongs to the information set I⊆[N] if $w_{i}⩾d$. Otherwise, *i* belongs to the frozen set F=[N]∖I. A codeword $c=[c_{0},c_{1},\ldots ,c_{N-1}]$ of RM(m,r) is encoded with a *N*-length binary vector $\mu =[\mu_{0},\mu_{1},\ldots ,\mu_{N-1}]$ as $c=\mu H_{2^{m}}$, where the *K* elements of μ with indices in I convey the data bits, while the remaining N−K elements are set to 0.

### 2.3. SC and SCL Decoding

**Figure 1:**
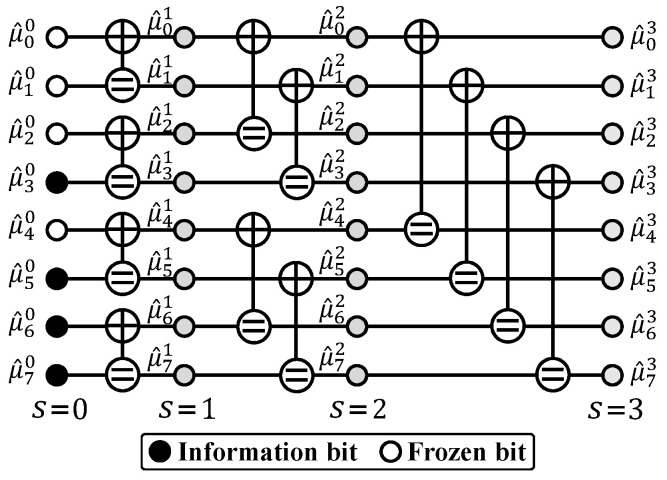
FG representation of the RM(3,1) code. White and black circles at the 0-th stage represent frozen bits and information bits, respectively.

Based on the HT definition, RM codes and polar codes share a common factor graph (FG) representation [3]. As depicted in Figure 1, an RM(*m*,*r*) code can be represented by an (m+1)-stage FG. In this structure, the 0-th stage corresponds to the message vector μ, while the *m*-th stage represents the codeword c. Furthermore, let $y^{s}$ denote the log-likelihood ratio (LLR) messages and ${\hat{\mu }}^{s}$ represent the hard decisions at the *s*-th stage. The successive cancellation (SC) decoding initializes $y^{m}$ using the channel LLRs $y^{ch}$ and recursively computes $y^{s}$ in a right-to-left manner as

(2) $$\begin{array}{cc} y_{i}^{s-1} & =f(y_{i}^{s},y_{i+2^{s-1}}^{s}), \end{array}$$

(3) $$\begin{array}{cc} y_{i+2^{s-1}}^{s-1} & =g(y_{i}^{s},y_{i+2^{s-1}}^{s},{\hat{\mu }}_{i}^{s-1}), \end{array}$$

where the subscript denotes the bit index, and the functions *f* and *g* are defined as

(4) $$\begin{array}{cc} f(a,b) & =\ln(\frac{e^{a+b}+1}{e^{a}+e^{b}}), \end{array}$$

(5) $$\begin{array}{cc} g(a,b,\mu ) & =(1-2\mu )a+b. \end{array}$$

The recursion terminates at the 0-th stage, where either an information bit estimate is made or the frozen bit value 0 is returned. Then, the hard decisions are propagated from left to right as

(6) $$\begin{array}{cc} {\hat{\mu }}_{i}^{s+1} & ={\hat{\mu }}_{i}^{s}⊕{\hat{\mu }}_{i+2^{s}}^{s}, \end{array}$$

(7) $$\begin{array}{cc} {\hat{\mu }}_{i+2^{s}}^{s+1} & ={\hat{\mu }}_{i+2^{s}}^{s}. \end{array}$$

Although SC decoding exhibits low computational complexity, its error-correction performance is suboptimal for short to moderate code lengths. To address this limitation, SCL decoding has been introduced to improve the performance of SC decoding [19]. In SCL decoding, both possible values (0 and 1) are considered for each information bit estimate ${\hat{\mu }}_{i}^{0}$ (where i∈I), leading to a path splitting mechanism that doubles the number of decoding paths after each such decision. To manage the exponential increase in the number of paths, a path metric (PM) is utilized to retain only the *L* most probable decoding paths after each information bit is decoded. Let ${\mathrm{PM}}_{i}^{\varepsilon }$ represent the path metric and ${\hat{\mu }}_{\left[0,i\right]}^{0,\varepsilon }$ denote the partial bit estimates of the ε-th path after decoding the *i*-th bit at the 0-th stage of FG. The path metric ${\mathrm{PM}}_{i}^{\varepsilon }$ is defined as follows:

(8) $${\mathrm{PM}}_{i}^{\varepsilon }=-\ln\left(\mathrm{Pr}\left[{\hat{\mu }}_{\left[0,i\right]}^{0,\varepsilon }|y^{ch}\right]\right).$$

After decoding each information bit, only the *L* paths with the smallest PM values are retained for further decoding. Once the decoding ends, the path with the smallest PM is chosen as the final output.

### 2.4. Automorphism Ensemble Decoding

An automorphism π of an *N*-length code C is a permutation on {0,1,…,N−1}, which satisfies π(c)∈C,∀c∈C. The automorphism group Aut(C) of a code C is the set containing all automorphisms of the code. For RM codes, the automorphism group is known to be the general affine group GA(m) [22], which consists of all affine bijections over $F_{2}^{m}$. An affine bijection is defined as the mapping $z^{*}=A_{m}z+b$, where $A_{m}\in F_{2}^{m\times m}$ is an invertible matrix, and $b\in F_{2}^{m\times 1}$ is an arbitrary vector. Here, z and $z^{*}$ are the *m*-bit binary representations of the code bit positions *i* and π(i), respectively.

**Figure 2:**
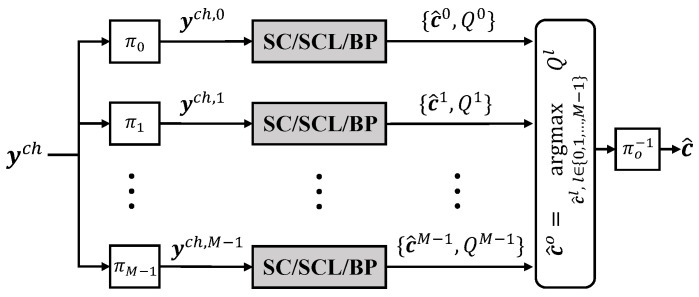
Framework of AED with various constituent decoders.

As illustrated in Figure 2, AED employs *M* randomly sampled permutations from the automorphism group of RM codes, denoted as $\pi_{l}$ for l∈{0,1,2,…,M−1}. For each permutation $\pi_{l}$, the channel LLRs $y^{ch}$ are interleaved to a new LLR vector $y^{ch,l}$, which is then decoded by a constituent decoder $D_{l}$ to produce a codeword estimate ${\hat{c}}^{l}$. The constituent decoder can be an SC, SCL, or BP decoder. Let $Q^{l}$ represent the metric corresponding to the codeword estimate of $D_{l}$, which could be a least-squares metric [21] or the LLR-based PM [32] if using an SC or SCL constituent decoder. Based on the metrics from the *M* constituent decoders, the best codeword estimate is selected. Let $\pi_{o}$ denote the permutation that yields the best metric. Finally, AED generates the output $\hat{c}$ by deinterleaving ${\hat{c}}^{o}$ with $\pi_{o}$.

AED leverages the symmetry of RM codes, specifically through the use of automorphisms, to achieve superior decoding performance. By considering multiple permutations of the corrupted codeword, AED effectively explores different noise realizations, increasing the possibility of finding a decodable version. Since most decoders are linear and influenced only by channel noise, using automorphisms is equivalent to permuting the noise [21]. This diversity in AED helps mitigate the effects of channel noise, as different permutations can lead to decodable error patterns [20]. Thus, by employing a reasonable number of automorphisms with appropriate constituent decoders, AED offers enhanced decoding performance for RM codes with practical complexity.

### 2.5. SC- and SCL-Based AED Using Factor Graph Permutation Group

In this study, we only consider a subset of the full automorphism group GA(m) of RM codes, denoted as P(m), which is the FG permutation group (FGPG). The FGPG consists of *m*! permutations that correspond to FG stage shuffles [34]. Specifically, each affine bijection in the FGPG uses an $A_{m}$, which is an m×m permutation matrix $P_{m}$ with only one 1 in each row and column and b=0. In the following, we will use the permutation notation $i^{*}=\pi^{N}\left(i\right)$ and its permutation matrix representation $P_{m}$ interchangeably. Additionally, we adopt SC and SCL decoders as the constituent decoder of AED and use the LLR-based PM to select the best codeword estimate. When using the SC constituent decoder, we denote the LLR messages and hard decisions at the *s*-th stage of FG for $D_{l}$ as $y^{s,l}$ and ${\hat{\mu }}^{s,l}$, respectively. Additionally, ${\hat{y}}_{\left[0,i\right]}^{s,l}$ and ${\hat{\mu }}_{\left[0,i\right]}^{s,l}$ represent the first i+1 LLR messages and bit estimates at the *s*-th stage of FG for $D_{l}$, respectively. For the SCL constituent decoder with a list size *L*, ${\hat{\mu }}^{0,\sigma ,l}$ denotes the bit estimates of the σ-th final decoding path of $D_{l}$, where σ∈{0,1,…,L−1}, while ${\hat{\mu }}_{\left[0,i\right]}^{0,\sigma ,l}$ represents the first i+1 partial bit estimates of the σ-th currently surviving decoding path of $D_{l}$ during runtime.

For the AED that has an automorphism ensemble size *M* and uses the SC constituent decoder, denoted as AE-*M*-SC, $Q^{l}$ is defined as

(9) $$Q^{l}=-\ln\left(\mathrm{Pr}\left[{\hat{\mu }}^{0,l}|y^{ch,l}\right]\right).$$

For the AED that uses the SCL constituent decoder with a list size of *L*, denoted as AE-*M*-SCL-*L*, $Q^{l}$ is defined as

(10) $$Q^{l}=\max_{\sigma \in \{0,1,\ldots ,L-1\}}\left(-\ln\left(\mathrm{Pr}\left[{\hat{\mu }}^{0,\sigma ,l}|y^{ch,l}\right]\right)\right).$$

In addition, we define a partial constituent metric (PCM) of the constituent decoders, denoted as $Q_{i}^{l}$. When using the SC constituent decoder, $Q_{i}^{l}$ is calculated as

(11) $$Q_{i}^{l}=-\ln\left(\mathrm{Pr}\left[{\hat{\mu }}_{\left[0,i\right]}^{0,l}|y^{ch,l}\right]\right),$$

which is essentially the partial PM of SC decoding. When using the SCL constituent decoder, $Q_{i}^{l}$ is given by

(12) $$Q_{i}^{l}=\max_{\sigma \in \{0,1,\ldots ,L-1\}}\left(-\ln\left(\mathrm{Pr}\left[{\hat{\mu }}_{\left[0,i\right]}^{0,\sigma ,l}|y^{ch,l}\right]\right)\right).$$

## 3. Subcode Estimate Convergence-Aided Path Pruning

In this section, we first present a Plotkin construction-based partitioning of the information set of RM codes. Combined with a uniform partitioning of FGPG, we prove that when using the permutations from the same subgroup of FGPG, the element distribution of the partitioned information set exhibits a permutation-invariance (PI) feature. This PI property enables a subcode estimate convergence (SEC) phenomenon in the SC- or SCL-based AED for RM codes. Observing that a strong SEC typically indicates a low noise level, where the full decoding capacity of AED is often unnecessary, we develop an SEC-aided path pruning technique that deactivates a large portion of constituent decoders when the intensity of SEC exceeds a preset threshold. The proposed SEC-aided path pruning performs a runtime adjustment of the decoding complexity without sacrificing the error-correction performance.

### 3.1. Subcode-Based Partitioning of Information Set

As described in Section 2.2, the information set $I^{m,r}$ of the RM(*m*,*r*) code can be obtained via a straightforward row weight-based index selection of the HT matrix $H_{2^{m}}$. For $H_{2^{m}}$, the weight of *i*-th row, $w_{i}$, can be calculated from the weight of the binary representation (BR) of *i*, where $0\leq i<2^{m}$. Specifically, let $z_{m}^{i}={\left[z_{0}^{i},z_{1}^{i},\ldots ,z_{m-1}^{i}\right]}^{⊤}$ denote the *m*-bit BR of *i* and $\phi \left(z_{m}^{i}\right)=\sum_{t=0}^{m-1}z_{t}^{i}$ denote the weight of $z_{m}^{i}$. We have $w_{i}=2^{\phi (z_{m}^{i})}$. Thus, $I^{m,r}$ is equivalently defined as [21]

(13) $$I^{m,r}=\left\{i|\phi \left(z_{m}^{i}\right)⩾m-r,0\leq i<2^{m}\right\}.$$

According to the Plotkin construction of RM codes [35], a codeword c∈RM(m,r) can be divided into two ($2^{m-1}$)-length subvectors u and u+v, where u∈RM(m−1,*r*), v∈RM(m−1,r−1) and c=(u,u+v). Following this recursion-style code decomposition, we partition $I^{m,r}$ into two parts as $I^{m,r}=\left[I_{v}^{m,r},I_{u}^{m,r}\right]$, where $I_{v}^{m,r}$ and $I_{u}^{m,r}$ are defined as

(14) $$\begin{array}{cc} I_{v}^{m,r}= & \{i|\phi \left(z_{m}^{i}\right)⩾m-r,0\leq i<2^{m-1}\}, \end{array}$$

(15) $$\begin{array}{cc} I_{u}^{m,r}= & \{i|\phi \left(z_{m}^{i}\right)⩾m-r,2^{m-1}\leq i<2^{m}\}. \end{array}$$

To further relate $I_{v}^{m,r}$ and $I_{u}^{m,r}$ to the v and u subcodes, we introduce two operations applied to binary vectors, denoted as bit deletion (BD) and bit insertion (BI).

**Definition** **1.** *With 0≤τ<m, BD-τ of $z_{m}^{i}$, denoted as $B_{-}\left(z_{m}^{i},\tau \right)$, deletes $z_{\tau }^{i}$ in $z_{m}^{i}$, resulting in an (m−1)-bit vector, while BI-(τ,b) of $z_{m}^{i}$, denoted as $B_{+}\left(z_{m}^{i},\tau ,b\right)$, inserts a bit b after $z_{\tau }^{i}$, generating an (m+1)-bit vector. When applied to multiple binary vectors, the same BD or BI operation is performed on each vector.*

Leveraging the BD and BI operations above, $I^{m,r}$ can be formulated with the information sets of u and v subcodes as follows.

**Theorem** **1.** *$I_{v}^{m,r}=B_{+}\left(I^{m-1,r-1},m-1,0\right)$ and $I_{u}^{m,r}=B_{+}\left(I^{m-1,r},m-1,1\right)$.*

**Proof** **of** **Theorem** **1.** As any element $i\in I_{v}^{m,r}$ has $z_{m-1}^{i}=0$, and any element $i\in I_{u}^{m,r}$ has $z_{m-1}^{i}=1$, according to Equations (14) and (15), we have

(16) $$\begin{array}{cc} B_{-}\left(I_{v}^{m,r},m-1\right)= & \{i|\phi \left(z_{m-1}^{i}\right)⩾m-r,0\leq i<2^{m-1}\},\\ = & \{i|\phi \left(z_{m-1}^{i}\right)⩾\left(m-1\right)-\left(r-1\right),0\leq i<2^{m-1}\},\\ = & I^{m-1,r-1}, \end{array}$$

(17) $$\begin{array}{cc} B_{-}\left(I_{u}^{m,r},m-1\right)= & \{i|\phi \left(z_{m-1}^{i}\right)⩾m-r-1,0\leq i<2^{m-1}\},\\ = & \{i|\phi \left(z_{m-1}^{i}\right)⩾\left(m-1\right)-r,0\leq i<2^{m-1}\},\\ = & I^{m-1,r}. \end{array}$$

Therefore, $I_{v}^{m,r}=B_{+}\left(I^{m-1,r-1},m-1,0\right)$, and $I_{u}^{m,r}=B_{+}\left(I^{m-1,r},m-1,1\right)$.    □

### 3.2. Permutation Invariance of Information Set Partitioning

For any permutation matrix $P_{m}\in P\left(m\right)$, it has the following property when used for the affine bijections of the FGPG.

**Property** **1.** Let $p_{a,b}$ denote the a-th element of the b-th column of $P_{m}\in P\left(m\right)$, where 0≤a<m and 0≤b<m. For two m-bit indices i and $i^{*}$, if $z_{m}^{i^{*}}=P_{m}z_{m}^{i}$ and $p_{a,b}=1$, we have $B_{-}\left(z_{m}^{i^{*}},a\right)=P_{m}^{/(a,b)}B_{-}\left(z_{m}^{i},b\right)$, where $P_{m}^{/(a,b)}$ denotes the (m−1)×(m−1) matrix derived by deleting the a-th row and the b-th column of $P_{m}$.

**Proof** **of** **Property** **1.** By performing m−1−a row-wise swaps and m−1−b column-wise swaps on $P_{m}$, the element $p_{a,b}$ can be shifted to the bottom-right corner, resulting in a transformed matrix $P_{m}^{\prime }$ given by

(18) $$P_{m}^{\prime }=\left[\begin{array}{cc} P_{m}^{/(a,b)} & 0\\ 0 & 1 \end{array}\right].$$

Additionally, by performing m−1−a element-wise swaps on $z_{m}^{i^{*}}$ and m−1−b element-wise swaps on $z_{m}^{i}$, corresponding to the row-wise and column-wise swaps on $P_{m}$, respectively, we obtain

(19) $$\left[\begin{array}{c} B_{-}\left(z_{m}^{i^{*}},a\right)\\ z_{a}^{i^{*}} \end{array}\right]=\left[\begin{array}{cc} P_{m}^{/(a,b)} & 0\\ 0 & 1 \end{array}\right]\cdot \left[\begin{array}{c} B_{-}\left(z_{m}^{i},b\right)\\ z_{b}^{i} \end{array}\right].$$

Consequently, we have $B_{-}\left(z_{m}^{i^{*}},a\right)=P_{m}^{/(a,b)}B_{-}\left(z_{m}^{i},b\right)$.    □

**Figure 3:**
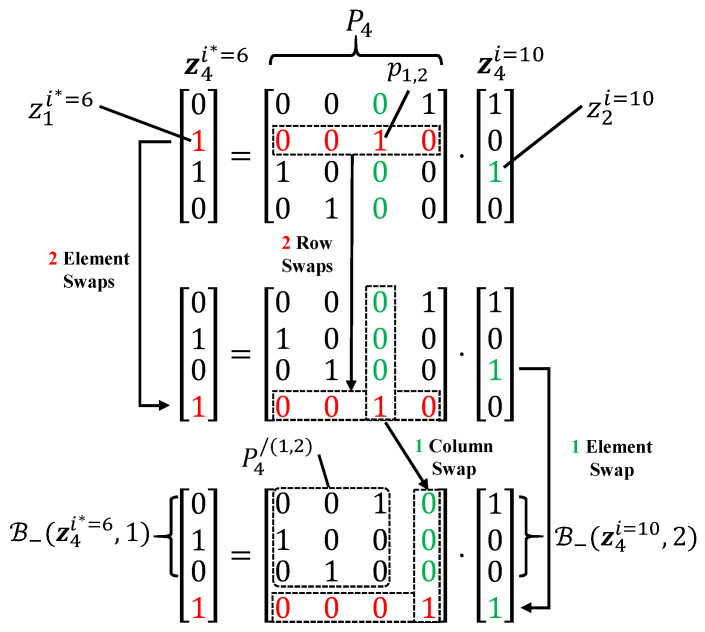
Demonstration of Property 1 using a permutation matrix $P_{4}\in P\left(4\right)$ with $p_{1,2}=1$ and two indices i=10 and $i^{*}=6$.

Figure 3 illustrates an example of Property 1, where a permutation matrix $P_{4}\in P\left(4\right)$ with $p_{1,2}=1$ and two indices i=10 and $i^{*}=6$ are used for demonstration.

As each automorphism from the FGPG corresponds to a unique permutation matrix $P_{m}$ that performs bit significance permutation (BSP) to the BR of the indices, it incurs no variation in the Hamming weight before and after use. Thus, when applying the permutation matrix $P_{m}$ to all the elements of the information set $I^{m,r}$, the transformed index vector has the following property.

**Property** **2.** *Let $J_{P_{m}}^{m,r}=\left[j_{0},j_{1},\ldots ,j_{K-1}\right]$ represent the index vector transformed from $I^{m,r}=\left[i_{0},i_{1},\ldots ,i_{K-1}\right]$ with $P_{m}\in P\left(m\right)$. This transformation is denoted as $J_{P_{m}}^{m,r}=P_{m}\left(I^{m,r}\right)$, where $z_{m}^{j_{t}}=P_{m}z_{m}^{i_{t}}$ with 0≤t<K. There exists a permutation $\pi_{P_{m}}^{K}$ on [K] that satisfies $J_{P_{m}}^{m,r}=\pi_{P_{m}}^{K}\left(I^{m,r}\right)$.*

**Proof** **of** **Property** **2.** Since $J_{P_{m}}^{m,r}=P_{m}\left(I^{m,r}\right)$ is a linear transformation defined by an invertible *m*-dimensional permutation matrix $P_{m}$, $J_{P_{m}}^{m,r}$ contains the same *K* unique elements as $I^{m,r}$, each with a Hamming weight of at least m−r. Thus, $J_{P_{m}}^{m,r}$ and $I^{m,r}$ consist of identical elements but arranged in a different order. The one-to-one element mapping between $J_{P_{m}}^{m,r}$ and $I^{m,r}$ can be characterized by a permutation $\pi_{P_{m}}^{K}$ on [K].    □

As the BSP can be viewed as the stage shuffles of FG [34], the permuted codeword $\pi^{N}\left(c\right)$ can be encoded with the permuted vector $\pi^{N}\left(\mu \right)$ as

(20) $$\pi^{N}\left(c\right)=\pi^{N}\left(\mu \right)H_{2^{m}}.$$

Property 2 demonstrates that ${\left(\pi^{N}\left(\mu \right)\right)}_{I^{m,r}}=\mu_{J_{P_{m}}^{m,r}}=\pi_{P_{m}}^{K}\left(\mu_{I^{m,r}}\right)$. This indicates that the *K* information bits are permuted within the *K* bit positions of $I^{m,r}$ using $\pi_{P_{m}}^{K}$. In other words, $\mu_{J_{P_{m}}^{m,r}}$ corresponds to the *K* information bits of the RM codeword $\pi^{N}\left(c\right)$. Similar to $I^{m,r}$, we partition $J_{P_{m}}^{m,r}$ into two parts as $J_{P_{m}}^{m,r}=\left[J_{v,P_{m}}^{m,r},J_{u,P_{m}}^{m,r}\right]$, where $J_{v,P_{m}}^{m,r}=P_{m}\left(I_{v}^{m,r}\right)$, and $J_{u,P_{m}}^{m,r}=P_{m}\left(I_{u}^{m,r}\right)$. Further, we divide P(m) into *m* partial permutation groups (PPGs) as follows.

**Definition** **2.** *The i-th PPG of P(m), denoted as G(m,i) with 0≤i<m, contains (m−1)! permutations with corresponding $P_{m}$ exhibiting $p_{i,m-1}=1$.*

With the uniform partitioning of P(m) defined above, we have the following property.

**Property** **3.** *For any two permutations from G(m,i) that correspond to the permutation matrixes $G_{m,\alpha }$ and $G_{m,\beta }$, respectively, there exists a permutation $\pi_{\alpha ,\beta }^{K_{v}}$ on $\left[K_{v}\right]$ that satisfies $J_{v,G_{m,\alpha }}^{m,r}=\pi_{\alpha ,\beta }^{K_{v}}\left(J_{v,G_{m,\beta }}^{m,r}\right)$ and a permutation $\pi_{\alpha ,\beta }^{K_{u}}$ on $\left[K_{u}\right]$ that satisfies $J_{u,G_{m,\alpha }}^{m,r}=\pi_{\alpha ,\beta }^{K_{u}}\left(J_{u,G_{m,\beta }}^{m,r}\right)$, where Kv=∑i=0r−1m−1i, and Ku=∑i=0rm−1i.*

**Proof** **of** **Property** **3.** For a permutation matrix $G_{m}$ which corresponds to an automorphism from G(m,i), we have

(21) $$\begin{array}{cc} J_{v,G_{m}}^{m,r}= & G_{m}\left(I_{v}^{m,r}\right)\\ = & G_{m}\left(B_{+}\left(I^{m-1,r-1},m-1,0\right)\right)\\ = & B_{+}\left(G_{m}^{/(i,m-1)}\left(I^{m-1,r-1}\right),i,0\right)\\ = & B_{+}\left(J_{G_{m}^{/(i,m-1)}}^{m-1,r-1},i,0\right). \end{array}$$

Similarly, we have

(22) $$\begin{array}{cc} J_{u,G_{m}}^{m,r}= & G_{m}\left(I_{u}^{m,r}\right)\\ = & G_{m}\left(B_{+}\left(I^{m-1,r},m-1,1\right)\right)\\ = & B_{+}\left(G_{m}^{/(i,m-1)}\left(I^{m-1,r}\right),i,1\right)\\ = & B_{+}\left(J_{G_{m}^{/(i,m-1)}}^{m-1,r},i,1\right). \end{array}$$

For the permutation matrixes $G_{m,\alpha }$ and $G_{m,\beta }$, $G_{m,\alpha }^{/(i,m-1)}$ and $G_{m,\beta }^{/(i,m-1)}$ are two (m−1)-dimensional permutation matrixes from P(m−1). Therefore, according to Property 2, there exists a permutation $\pi_{\alpha ,\beta }^{K_{v}}$ on $\left[K_{v}\right]$ that gives

(23) $$J_{G_{m,\alpha }^{/(i,m-1)}}^{m-1,r-1}=\pi_{\alpha ,\beta }^{K_{v}}\left(J_{G_{m,\beta }^{/(i,m-1)}}^{m-1,r-1}\right)$$

and a permutation $\pi_{\alpha ,\beta }^{K_{u}}$ on $\left[K_{u}\right]$ that satisfies

(24) $$J_{G_{m,\alpha }^{/(i,m-1)}}^{m-1,r}=\pi_{\alpha ,\beta }^{K_{u}}\left(J_{G_{m,\beta }^{/(i,m-1)}}^{m-1,r}\right).$$

Equations (21) and (22) show that $J_{v,G_{m}}^{m,r}$ is formulated by inserts 0 after the *i*-th bit of each element in $J_{G_{m}^{/(i,m-1)}}^{m-1,r-1}$, and $J_{u,G_{m}}^{m,r}$ is formulated by inserts 1 after the *i*-th bit of each element in $J_{G_{m}^{/(i,m-1)}}^{m-1,r}$. Thus, we have

(25) $$\begin{array}{cc} J_{v,G_{m,\alpha }}^{m,r} & =\pi_{\alpha ,\beta }^{K_{v}}\left(J_{v,G_{m,\beta }}^{m,r}\right), \end{array}$$

(26) $$\begin{array}{cc} J_{u,G_{m,\alpha }}^{m,r} & =\pi_{\alpha ,\beta }^{K_{u}}\left(J_{u,G_{m,\beta }}^{m,r}\right). \end{array}$$

   □

Equations (25) and (26) indicate that for all the permutations from the same PPG, $J_{P_{m}}^{m,r}$ exhibits identical subcode-based element partitioning. The only distinction lies in the order of elements within $J_{v,P_{m}}^{m,r}$ and $J_{u,P_{m}}^{m,r}$.

The origin of the PI property lies in the fact that the automorphisms from the FGPG correspond to BSP of the binary representation of the indices. When we divide the indices from 0 to N−1 into two equal halves, if two automorphisms from the FGPG move the MSB of the first $\frac{N}{2}$ indices to the same new bit position, and if we ignore this position, the remaining m−1 bits of each index also undergo a BSP. Since the MSB of the original first $\frac{N}{2}$ indices is 0, the first $\frac{N}{2}$ indices, after being permuted by these two automorphisms, can be permuted to each other through a one-to-one mapping. The same logic applies to the second $\frac{N}{2}$ indices, whose MSB is 1. In other words, the two automorphisms partition the *N* indices into the same two parts, despite the elements of each part being arranged in a distinct order. Moreover, since the BSP does not affect the Hamming weight, the elements of the information set contained in each half part are also the same for these two automorphisms, which also only differ in element order. Thus, the PI property of information set partitioning exists between automorphisms that move the MSB to the same new bit position, which is the underlying logic for dividing the FGPG into *m* PPGs as defined in Definition 2.

### 3.3. Subcode-Based Partial Constituent Metric Convergence

For the AE-*M*-SC decoder, we define a subcode-based PCM (SPCM) corresponding to the v subcode for each SC constituent decoder $D_{l}$, denoted as $Q_{v}^{l}$. Let $P_{m}^{l}$ denote the permutation matrix that corresponds to the permutation $\pi_{l}^{N}$ used by $D_{l}$. The SPCM $Q_{v}^{l}$ is given by

(27) $$Q_{v}^{l}=Q_{\frac{N}{2}-1}^{l}=-\ln\left(\mathrm{Pr}\left[{\hat{\mu }}_{\left[0,\frac{N}{2}-1\right]}^{0,l}|y^{ch,l}\right]\right),$$

where $\mu_{\left[0,\frac{N}{2}-1\right]}^{0,l}=\mu_{P_{m}^{l}\left(\left[0,\frac{N}{2}-1\right]\right)}$. For the rate-1 RM(m,m) code, $I_{v}^{m,m}=\left[0,\frac{N}{2}-1\right]$. According to Equations (23) and (25), for two SC constituent decoders $D_{\alpha },D_{\beta }$ using permutations $\pi_{\alpha },\pi_{\beta }\in G\left(m,i\right)$ that correspond to permutation matrixes $G_{m,\alpha },G_{m,\beta }$, there exists a permutation $\pi_{\alpha ,\beta }^{\frac{N}{2}}$ on $\left[\frac{N}{2}\right]$ that satisfies

(28) $$\begin{array}{cc} J_{G_{m,\alpha }^{/(i,m-1)}}^{m-1,m-1} & =\pi_{\alpha ,\beta }^{\frac{N}{2}}\left(J_{G_{m,\beta }^{/(i,m-1)}}^{m-1,m-1}\right), \end{array}$$

(29) $$\begin{array}{cc} J_{v,G_{m,\alpha }}^{m,m} & =\pi_{\alpha ,\beta }^{\frac{N}{2}}\left(J_{v,G_{m,\beta }}^{m,m}\right), \end{array}$$

which implies that

(30) $$\begin{array}{cc} \mu_{\left[0,\frac{N}{2}-1\right]}^{0,\alpha }= & \pi_{\alpha ,\beta }^{\frac{N}{2}}\left(\mu_{\left[0,\frac{N}{2}-1\right]}^{0,\beta }\right). \end{array}$$

Thus, the first $\frac{N}{2}$ bit estimates at the 0-th stage of FG that correspond to the v subcode could be identical for $D_{\alpha }$ and $D_{\beta }$ despite the different bit orders, particularly under low noise levels where most SC constituent decoders tend to decode the codeword correctly. When $D_{\alpha }$ and $D_{\beta }$ both decode the first $\frac{N}{2}$ bits at the 0-th stage of FG correctly, i.e., ${\hat{\mu }}_{\left[0,\frac{N}{2}-1\right]}^{0,\alpha }=\pi_{\alpha ,\beta }^{\frac{N}{2}}\left({\hat{\mu }}_{\left[0,\frac{N}{2}-1\right]}^{0,\beta }\right)$, it follows that $Q_{v}^{\alpha }=Q_{v}^{\beta }$ according to Equation (27), demonstrating a notable SPCM convergence (SPCMC) phenomenon between the SC constituent decoders.

Although we have demonstrated that the SPCMC likely occurs in the AE-*M*-SC decoder, the SPCM defined with Equation (27), which is essentially a partial PM of SC decoding, is not implementation-friendly. However, Equation (9) (or Equation (27)) can be expressed as [32]

(31) $$Q_{v}^{l}≜\sum_{t=0}^{\frac{N}{2}-1}\ln\left(1+e^{-\left(1-2{\hat{\mu }}_{t}^{0,l}\right)\cdot y_{t}^{0,l}}\right),$$

which is usually approximated as

(32) $$Q_{v}^{l}=-\sum_{t\in I_{v}^{m,r}}\min\left\{0,\left(1-2{\hat{\mu }}_{t}^{0,l}\right)\cdot y_{t}^{0,l}\right\}.$$

We further prove that using the hardware-friendly min{.,.} approximation of the *f* function in Equation (4) and the simplified version of SPCM in Equation (32), the SPCMC still occurs between the SC constituent decoders that recover the codeword with the permutations from the same PPG.

When the *f* function adopts the min{.,.} approximation as

(33) f(a,b)≈sign(a)sign(b)min(|a|,|b|),

Equation (32) is equivalent to [31]

(34) $$Q_{v}^{l}=-\sum_{t=0}^{\frac{N}{2}-1}\min\left\{0,\left(1-2{\hat{\mu }}_{t}^{m-1,l}\right)\cdot y_{t}^{m-1,l}\right\}.$$

If rewritten into a vector form, Equation (34) is expressed as

(35) $$Q_{v}^{l}=-\vec{\sum }\vec{\min}\left\{0,\left(1-2\cdot {\hat{\mu }}_{\left[0,\frac{N}{2}-1\right]}^{m-1,l}\right)\cdot y_{\left[0,\frac{N}{2}-1\right]}^{m-1,l}\right\},$$

where all the operations are applied element-wise, and 0 and 1 represent $\frac{N}{2}$-length all-zero and all-one vectors, respectively. Additionally, we define a $\frac{N}{2}$-length reference vector $\mu^{\mathrm{REF}}=\mu_{B_{+}\left(I^{m-1,m-1},i,0\right)}$. According to Equation (21), we have

(36) $$\begin{array}{cc} \mu_{\left[0,\frac{N}{2}-1\right]}^{0,\alpha } & =\mu_{B_{+}\left(J_{G_{m,\alpha }^{/(i,m-1)}}^{m-1,m-1},i,0\right)}, \end{array}$$

(37) $$\begin{array}{cc} \mu_{\left[0,\frac{N}{2}-1\right]}^{0,\beta } & =\mu_{B_{+}\left(J_{G_{m,\beta }^{/(i,m-1)}}^{m-1,m-1},i,0\right)}. \end{array}$$

As $\mu_{\left[0,\frac{N}{2}-1\right]}^{0,\alpha }$ and $\mu_{\left[0,\frac{N}{2}-1\right]}^{0,\beta }$ can be obtained from $\mu^{\mathrm{REF}}$ via permutations on $\left[\frac{N}{2}\right]$ which correspond to permutation matrices $G_{m,\alpha }^{/(i,m-1)}$ and $G_{m,\beta }^{/(i,m-1)}$, respectively, we have

(38) $$\mu_{\left[0,\frac{N}{2}-1\right]}^{0,\alpha }\cdot H_{2^{m-1}}=\pi_{\alpha ,\beta }^{\frac{N}{2}}\left(\mu_{\left[0,\frac{N}{2}-1\right]}^{0,\beta }\cdot H_{2^{m-1}}\right).$$

When the two SC constituent decoders $D_{\alpha }$ and $D_{\beta }$ both recover the codeword, i.e., ${\hat{\mu }}_{\left[0,\frac{N}{2}-1\right]}^{0,\alpha }=\mu_{\left[0,\frac{N}{2}-1\right]}^{0,\alpha }$, and ${\hat{\mu }}_{\left[0,\frac{N}{2}-1\right]}^{0,\beta }=\mu_{\left[0,\frac{N}{2}-1\right]}^{0,\beta }$, their partial sums at the (m−1)-th stage of FG will show ${\hat{\mu }}_{\left[0,\frac{N}{2}-1\right]}^{m-1,\alpha }=\pi_{\alpha ,\beta }^{\frac{N}{2}}\left({\hat{\mu }}_{\left[0,\frac{N}{2}-1\right]}^{m-1,\beta }\right)$. Furthermore, let $G_{m,l}$ denote the permutation matrix that corresponds to the permutation $\pi_{l}\in G\left(m,i\right)$ used in $D_{l}$. The LLR vector $y_{\left[0,\frac{N}{2}-1\right]}^{m-1,l}$ in Equation (35) is derived as

(39) $$\begin{array}{cc} y_{\left[0,\frac{N}{2}-1\right]}^{m-1,l}= & \vec{f}\left(y_{\left[0,\frac{N}{2}-1\right]}^{m,l},y_{\left[\frac{N}{2},N-1\right]}^{m,l}\right)\\ = & \vec{f}\left(y_{G_{m,l}\left(\left[0,\frac{N}{2}-1\right]\right)}^{ch},y_{G_{m,l}\left(\left[\frac{N}{2},N-1\right]\right)}^{ch}\right)\\ = & \vec{f}\left(y_{G_{m,l}\left(I_{v}^{m,m}\right)}^{ch},y_{G_{m,l}\left(I_{u}^{m,m}\right)}^{ch}\right)\\ = & \vec{f}\left(y_{G_{m,l}\left(B_{+}\left(I^{m-1,m-1},m-1,0\right)\right)}^{ch},y_{G_{m,l}\left(B_{+}\left(I^{m-1,m-1},m-1,1\right)\right)}^{ch}\right)\\ = & \vec{f}\left(y_{B_{+}\left(J_{G_{m,l}^{/(i,m-1)}}^{m-1,m-1},i,0\right)}^{ch},y_{B_{+}\left(J_{G_{m,l}^{/(i,m-1)}}^{m-1,m-1},i,1\right)}^{ch}\right). \end{array}$$

According to Equation (28), we have

(40) $$\begin{array}{c} y_{B_{+}\left(J_{G_{m,\alpha }^{/(i,m-1)}}^{m-1,m-1},i,0\right)}^{ch}=\pi_{\alpha ,\beta }^{\frac{N}{2}}\left(y_{B_{+}\left(J_{G_{m,\beta }^{/(i,m-1)}}^{m-1,m-1},i,0\right)}^{ch}\right), \end{array}$$

(41) $$\begin{array}{c} y_{B_{+}\left(J_{G_{m,\alpha }^{/(i,m-1)}}^{m-1,m-1},i,1\right)}^{ch}=\pi_{\alpha ,\beta }^{\frac{N}{2}}\left(y_{B_{+}\left(J_{G_{m,\beta }^{/(i,m-1)}}^{m-1,m-1},i,1\right)}^{ch}\right). \end{array}$$

Thus, we have $y_{\left[0,\frac{N}{2}-1\right]}^{m-1,\alpha }=\pi_{\alpha ,\beta }^{\frac{N}{2}}(y_{\left[0,\frac{N}{2}-1\right]}^{m-1,\beta }$). In summary, when $D_{\alpha }$ and $D_{\beta }$ both correctly decode the codeword, $Q_{v}^{\alpha }=Q_{v}^{\beta }$, as the permutation $\pi_{\alpha ,\beta }^{\frac{N}{2}}$ does not influence the element-wise computations in Equation (35) when applied to both the first half partial sums and LLRs at the (m−1)-th stage of the FG. Consequently, the SPCMC can still be observed between the SC constituent decoders when they successfully recover the codeword.

### 3.4. Subcode Estimate Convergence-Aided Path Pruning

Current state-of-the-art AE-SC decoders [21,31] employ randomly sampled permutations when only considering the FGPG. The analysis above suggests that by further partitioning these randomly selected permutations into a maximum of *m* groups based on their respective PPGs, the SC constituent decoders using permutations from the same PPG can generate identical first-half bit estimates corresponding to the v subcode, albeit in a different bit order. We refer to this phenomenon as subcode estimate convergence (SEC). Since SEC incurs SPCMC between the constituent decoders in the AE-SC decoder, it can be identified by detecting SPCMC at runtime.

Let $\lambda =[\lambda_{0},\lambda_{1},\ldots ,\lambda_{m-1}]$ denote the permutation distribution in the AE-*M*-SC decoder, where $\lambda_{t}$ denotes the number of permutations sampled from the PPG G(m,t), with $0\leq \lambda_{t}\leq M$, and $M=\sum_{t=0}^{m-1}\lambda_{t}$. Additionally, we use $\rho =[\rho_{0},\rho_{1},\ldots ,\rho_{m-1}]$ with $\rho_{t}\in \left\{0,1\right\}$ to characterize the SEC of the *t*-th group, where $\rho_{t}$ is 1, if all the $\lambda_{t}$ SC constituent decoders in the *t*-th group show SPCMC, and 0 otherwise. In other words, $\rho_{t}$ is used to indicate whether the SEC phenomenon has occurred within the *t*-th group during runtime. Additionally, we define an SEC intensity metric H across the *m* groups as

(42) $$H=\sum_{t=0}^{m-1}\rho_{t}.$$

**Figure 4:**
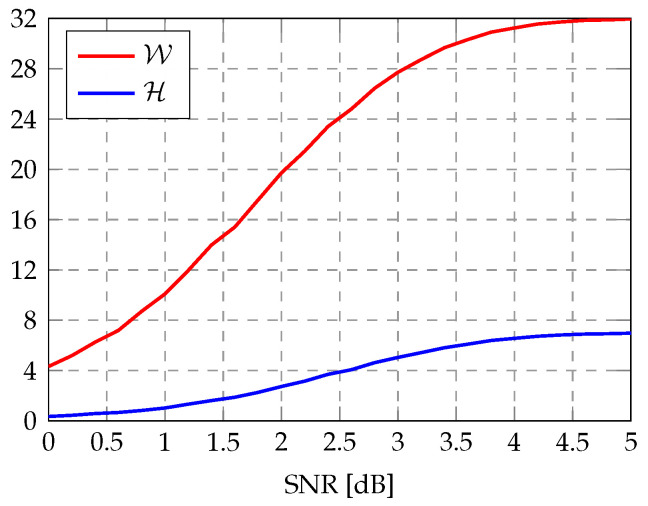
Statistical average values of W and H versus SNR in an AE32-SC decoder using λ=[4,4,4,5,5,5,5] for the RM(7,3) code.

Let W denote the number of SC constituent decoders that generate the best PM in the AE-SC decoding of one noisy codeword. Figure 4 illustrates the variation of average values of W and H against the signal-to-noise ratio (SNR), where the data are collected from an AE32-SC decoder for the RM(7,3) code using λ=[4,4,4,5,5,5,5]. As the SNR increases, W and H both increase, approaching M=32 and m=7 in the high SNR region, respectively. When the average H exceeds 4, more than $\frac{3}{4}$ of the SC constituent decoders share the best decoding output on average. This indicates that, in low noise conditions, the full decoding capacity of the AE-SC decoder is often unnecessary.

Since the average value of H monotonically increases with the SNR, we propose to use the SEC intensity as an indicator of the noise level and dynamically adjust the decoding complexity accordingly without compromising the performance. Specifically, we perform path pruning when the SEC intensity H is greater than or equal to a threshold $T=\lceil \frac{m}{2}\rceil$, resulting in an SEC-aided AE-SC (SEC-AE-SC) decoder. Let $S=\{s_{0},s_{1},\ldots ,s_{H-1}\}$ denote the set of indices of the groups that satisfy $\rho_{t}=1$ after all the *M* SC constituent decoders have generated the first half of bit estimates, where |S|=H. Let $\chi_{t}$ denote the minimum index of the SC constituent decoders in the *t*-th group. We then formulate a set of indices of SC constituent decoders with a cardinality of H, denoted as $X=\{\chi_{s_{0}},\chi_{s_{1}},\ldots ,\chi_{s_{H-1}}\}$. In our proposed SEC-AE-SC decoder, if H≥T, the *T* SC decoding paths corresponding to the first *T* indices in X are allowed to complete decoding, while the remaining M−T paths are terminated. If H<T, the SEC-AE-SC decoder reduces to the AE-SC decoder without path pruning. The formal description of the SEC-AE-SC decoder is provided in Algorithm 1. 
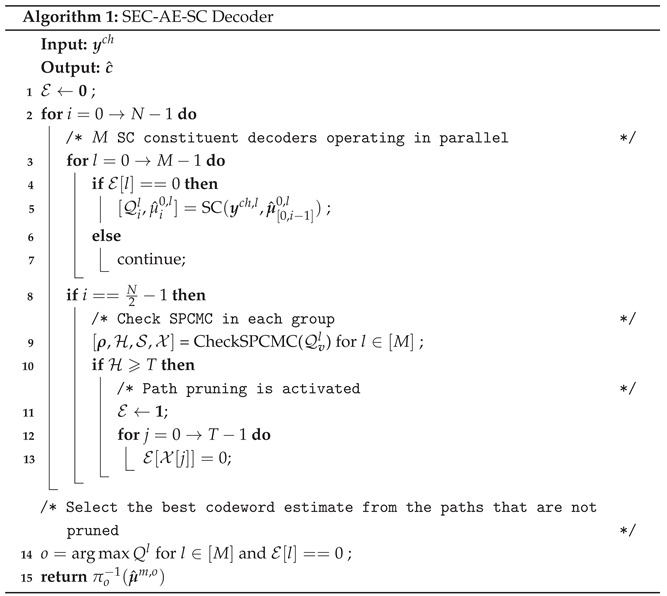


It is noteworthy that the proposed SEC-aided path pruning technique is also applicable to the case where the constituent decoder is an SCL decoder with a list size of *L*, resulting in an SEC-AE-*M*-SCL-*L* decoder. In this case, the SCL constituent decoder operates as an integrated unit, with its SPCM generated according to Equation (12) as

(43) $$Q_{v}^{l}=Q_{\frac{N}{2}-1}^{l}=\max_{\sigma \in \{0,1,\ldots ,L-1\}}\left(-\ln\left(\mathrm{Pr}\left[{\hat{\mu }}_{\left[0,\frac{N}{2}-1\right]}^{0,\sigma ,l}|y^{ch,l}\right]\right)\right).$$

Apart from the distinct derivation of the SPCM, the formulations of ρ, H, S, and X when utilizing the SCL constituent decoder remain identical to those when the constituent decoder is an SC decoder. If path pruning is activated by observing H≥T in the SEC-AE-*M*-SCL-*L* decoder, *T* SCL decoders corresponding to the first *T* indices in X are kept, while the remaining M−T SCL decoders are terminated.

The configuration of $\lambda_{t}$ should be as close as possible to $\frac{M}{m}$, meaning that the number of automorphisms selected from each PPG should be as consistent as possible to maximize randomness. For example, for a code length of 128 and M=32, since 32 cannot be evenly divided by m=7, a typical configuration for λ is λ=[4,4,4,5,5,5,5], which aims to ensure the uniformity of the selection as much as possible. The rationale behind this configuration of λ has two main points: Firstly, the selection of automorphisms is limited to the FGPG in this work, which inherently uses fewer noise realizations compared to the full automorphism group. The analysis of the PI property in information set partitioning indicates that the automorphisms sampled from the same PPG exhibit only local randomness. Therefore, the selection of automorphisms within the FGPG should be as random as possible to maximize the diversity gain of AED, without favoring any PPG, to achieve the best decoding performance that the FGPG can provide. Secondly, the proposed SEC-aided path pruning technique relies on the detection of SEC phenomena from *m* groups of constituent decoders. If the proportion of each group in the overall automorphism ensemble varies significantly, the possibility of detecting SEC in each group will differ, leading to distinct reliability in noise level evaluation. As a result, even if the SEC intensity threshold *T* is exceeded, the noise realization may still require the full decoding capability of AED, thus introducing a risk of performance loss through path pruning. Therefore, to ensure that each group provides similar noise evaluation capabilities when SEC is detected, the automorphism distribution of λ should be as uniform as possible, minimizing the performance degradation caused by path pruning.

### 3.5. Complexity of SEC-Aided AE Decoding

Let $C_{D}$ and $C_{\mathrm{AED}}$ denote the complexity of the constituent decoder D and the AE-*M*-D decoder, respectively. In this work, D could be an SC or an SCL-*L* decoder with a list size of *L*, where $C_{D}$ is O(NlogN) and O(LNlogN), respectively. Clearly, $C_{\mathrm{AED}}=M\times C_{D}$. Under the fully parallel (FP) AED implementation, the *M* constituent decoders operate in parallel. If the path pruning is triggered at runtime, the complexity of the proposed SEC-AE-*M*-D decoder, denoted as $C_{\mathrm{FP}\text{-}\mathrm{SEC}\text{-}\mathrm{AED}}^{H\geq T}$, is given by

(44) $$C_{\mathrm{FP}\text{-}\mathrm{SEC}\text{-}\mathrm{AED}}^{H\geq T}=\left(M-\left\lceil \frac{m}{2}\right\rceil \right)\times \frac{C_{D}}{2}+\left\lceil \frac{m}{2}\right\rceil \times C_{D}.$$

Let $C_{\mathrm{FP}\text{-}\mathrm{SEC}\text{-}\mathrm{AED}}^{H\geq T,\mathrm{norm}}$ denote the normalized complexity of the SEC-AE-*M*-D decoder with path pruning, which is expressed as

(45) $$C_{\mathrm{FP}\text{-}\mathrm{SEC}\text{-}\mathrm{AED}}^{H\geq T,\mathrm{norm}}=\frac{C_{\mathrm{FP}\text{-}\mathrm{SEC}\text{-}\mathrm{AED}}^{H\geq T}}{C_{\mathrm{AED}}}=\frac{1}{2}+\frac{\left\lceil \frac{m}{2}\right\rceil }{2M}.$$

Let $C_{\mathrm{FP}\text{-}\mathrm{SEC}\text{-}\mathrm{AED}}^{\mathrm{norm},\eta }$ represent the average normalized complexity of the FP-SEC-AE-*M*-D decoder at an SNR point η. Let $\delta_{\eta }$ denote the fraction of times that path pruning is activated at the SNR point η under the FP implementation. $C_{\mathrm{FP}\text{-}\mathrm{SEC}\text{-}\mathrm{AED}}^{\mathrm{norm},\eta }$ is calculated as

(46) $$C_{\mathrm{FP}\text{-}\mathrm{SEC}\text{-}\mathrm{AED}}^{\mathrm{norm},\eta }=\delta_{\eta }\times C_{\mathrm{FP}\text{-}\mathrm{SEC}\text{-}\mathrm{AED}}^{H\geq T,\mathrm{norm}}+\left(1-\delta_{\eta }\right).$$

Intuitively, $\delta_{\eta }$ increases with η because more constituent decoders are likely to recover the codeword at higher SNR levels, leading to a higher probability of observing SPCMC within each permutation group. Consequently, $C_{\mathrm{FP}\text{-}\mathrm{SEC}\text{-}\mathrm{AED}}^{\mathrm{norm},\eta }$ decreases, as the SNR increases. Equation (45) suggests that the proposed SEC-aided path pruning technique reduces the complexity of $C_{\mathrm{AED}}$ by no more than half when applied to the FP scheme. Additionally, Equation (46) indicates that, under the FP implementation, $C_{\mathrm{FP}\text{-}\mathrm{SEC}\text{-}\mathrm{AED}}^{H\geq T,\mathrm{norm}}$ represents the lower bound of the normalized complexity for the FP-SEC-AE-*M*-D decoder.

To further increase the complexity reduction, the SEC-AE-*M*-D decoder can be applied to a partially parallel (PP) implementation as follows: The constituent decoders of the first *T* groups are launched first. If $\rho_{t}=1$ for 0≤t<T, path pruning is activated to select *T* constituent decoders from the first *T* groups, while skipping the constituent decoders of the remaining m−T groups. If $\rho_{t}=0$ for some 0≤t<T, the constituent decoders of the remaining m−T groups are launched to evaluate H, similar to the FP implementation. If H≥T, path pruning is also activated. Let $C_{\mathrm{PP}\text{-}\mathrm{SEC}\text{-}\mathrm{AED}}^{T}$ and $C_{\mathrm{PP}\text{-}\mathrm{SEC}\text{-}\mathrm{AED}}^{T,\mathrm{norm}}$ denote the complexity and the normalized complexity of the PP-SEC-AE-*M*-D decoder when the path pruning is triggered within the first *T* groups. $C_{\mathrm{PP}\text{-}\mathrm{SEC}\text{-}\mathrm{AED}}^{T}$ is expressed as

(47) $$C_{\mathrm{PP}\text{-}\mathrm{SEC}\text{-}\mathrm{AED}}^{T}=\left(\sum_{t=0}^{\lceil \frac{m}{2}\rceil -1}\lambda_{t}-\left\lceil \frac{m}{2}\right\rceil \right)\frac{C_{D}}{2}+\left\lceil \frac{m}{2}\right\rceil C_{D},$$

while $C_{\mathrm{PP}\text{-}\mathrm{SEC}\text{-}\mathrm{AED}}^{T,\mathrm{norm}}$ is given by

(48) $$C_{\mathrm{PP}\text{-}\mathrm{SEC}\text{-}\mathrm{AED}}^{T,\mathrm{norm}}=\frac{C_{\mathrm{PP}\text{-}\mathrm{SEC}\text{-}\mathrm{AED}}^{T}}{C_{\mathrm{AED}}}=\frac{1}{2}\times \frac{\sum_{t=0}^{\lceil \frac{m}{2}\rceil -1}\lambda_{t}}{M}+\frac{\left\lceil \frac{m}{2}\right\rceil }{2M}.$$

Let $C_{\mathrm{PP}\text{-}\mathrm{SEC}\text{-}\mathrm{AED}}^{\mathrm{norm},\eta }$ denote the average normalized complexity of the PP-SEC-AE-*M*-D decoder at the SNR point η. Let $\delta_{\eta }^{T}$ denote the fraction of times that path pruning is activated within the first *T* groups at the SNR point η. $C_{\mathrm{PP}\text{-}\mathrm{SEC}\text{-}\mathrm{AED}}^{\mathrm{norm},\eta }$ is given by

(49) $$\begin{array}{c} C_{\mathrm{PP}\text{-}\mathrm{SEC}\text{-}\mathrm{AED}}^{\mathrm{norm},\eta }=\delta_{\eta }^{T}\times C_{\mathrm{PP}\text{-}\mathrm{SEC}\text{-}\mathrm{AED}}^{T,\mathrm{norm}}+\left(\delta_{\eta }-\delta_{\eta }^{T}\right)\times C_{\mathrm{FP}\text{-}\mathrm{SEC}\text{-}\mathrm{AED}}^{H\geq T,\mathrm{norm}}+\left(1-\delta_{\eta }\right). \end{array}$$

The PP scheme essentially performs the progressive detection of sufficiently intense SEC. When the constituent decoders of the first *T* groups exhibit an SEC intensity that triggers path pruning, the constituent decoders of the remaining m−T groups need not be launched. In such cases, path pruning is performed within the first *T* groups, and the PP scheme produces output equivalent to that of the FP implementation. Thus, the PP-SEC-AE-*M*-D decoder incurs no performance loss compared to the FP-SEC-AE-*M*-D decoder. As the SNR increases, path pruning is activated more frequently within the first *T* groups, causing $\delta_{\eta }^{T}$ to approach $\delta_{\eta }$. As a result, $C_{\mathrm{PP}\text{-}\mathrm{SEC}\text{-}\mathrm{AED}}^{\mathrm{norm},\eta }$ approaches $C_{\mathrm{PP}\text{-}\mathrm{SEC}\text{-}\mathrm{AED}}^{T,\mathrm{norm}}$, which is lower than the lower bound of $C_{\mathrm{FP}\text{-}\mathrm{SEC}\text{-}\mathrm{AED}}^{\mathrm{norm},\eta }$. However, the PP scheme introduces a 50% increase in worst-case decoding latency compared to the FP scheme.

## 4. Experimental Results and Discussion

In this section, the performance of the proposed SEC-AED is evaluated in terms of the block error rate (BLER) and complexity. Experiments are conducted over an additive white Gaussian noise (AWGN) channel, employing binary phase shift keying (BPSK) modulation and LLR demodulation. Four RM codes, namely RM(7,3), RM(7,4), RM(8,3), and RM(8,4), are considered to cover different code lengths and rates. For the RM(7,3) and RM(7,4) codes, we employ λ=[4,4,4,5,5,5,5] in the SEC-AED. For the RM(8,3) and RM(8,4) codes, we use λ=[4,4,4,4,4,4,4,4]. This λ configuration ensures the uniformity of permutation distribution, which maximizes the diversity gain of the AED.

### 4.1. Error-Correction Performance

**Figure 5:**
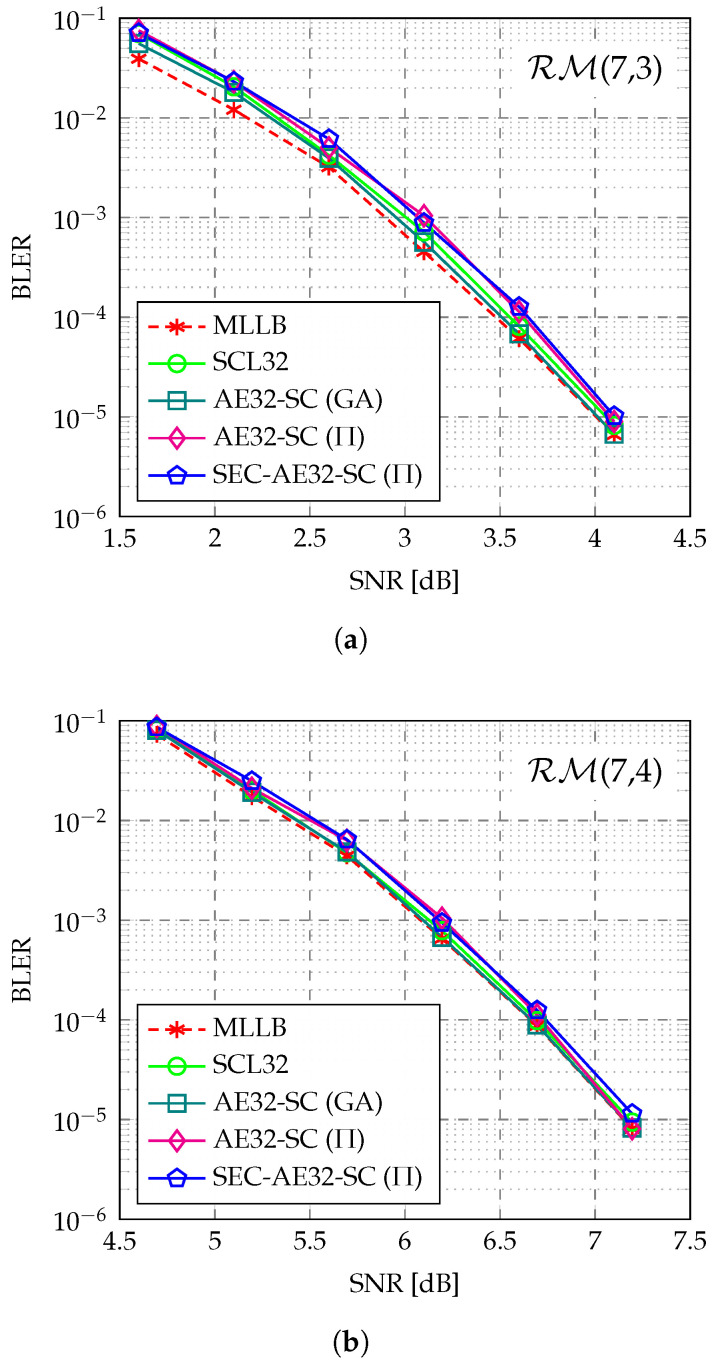
BLER comparison of MLLB [19], SCL32 decoder [32], AE32-SC (GA) decoder [21], AE32-SC (Π) decoder [31], and the proposed SEC-AE32-SC (Π) decoder for (**a**) RM(7,3) and (**b**) RM(7,4).

Figure 5 illustrates the BLER of different decoders for the RM(7,3) and RM(7,4) codes. The AED labeled as AED (GA) samples permutations from the full automorphism group, while the AED labeled as AED (Π) samples permutations from the FGPG. For reference, the ML lower bound (MLLB) [19], evaluated using the AE32-SC (GA) decoder, is also plotted. As depicted in Figure 5a,b, the SCL32 decoder and AE32-SC (GA) decoder exhibit negligible BLER loss compared to the MLLB for both RM(7,3) and RM(7,4) codes. The AE32-SC (Π) decoder also achieves near ML performance, with 0.14 dB and 0.09 dB losses relative to the MLLB at a BLER of $10^{-4}$ for the RM(7,3) and RM(7,4) codes, respectively. The proposed SEC-AE32-SC (Π) decoder demonstrates nearly identical BLER performance to the AE32-SC (Π) decoder, indicating that the proposed SEC-aided path pruning does not introduce noticeable performance degradation for decoding the RM(7,3) and RM(7,4) codes.

**Figure 6:**
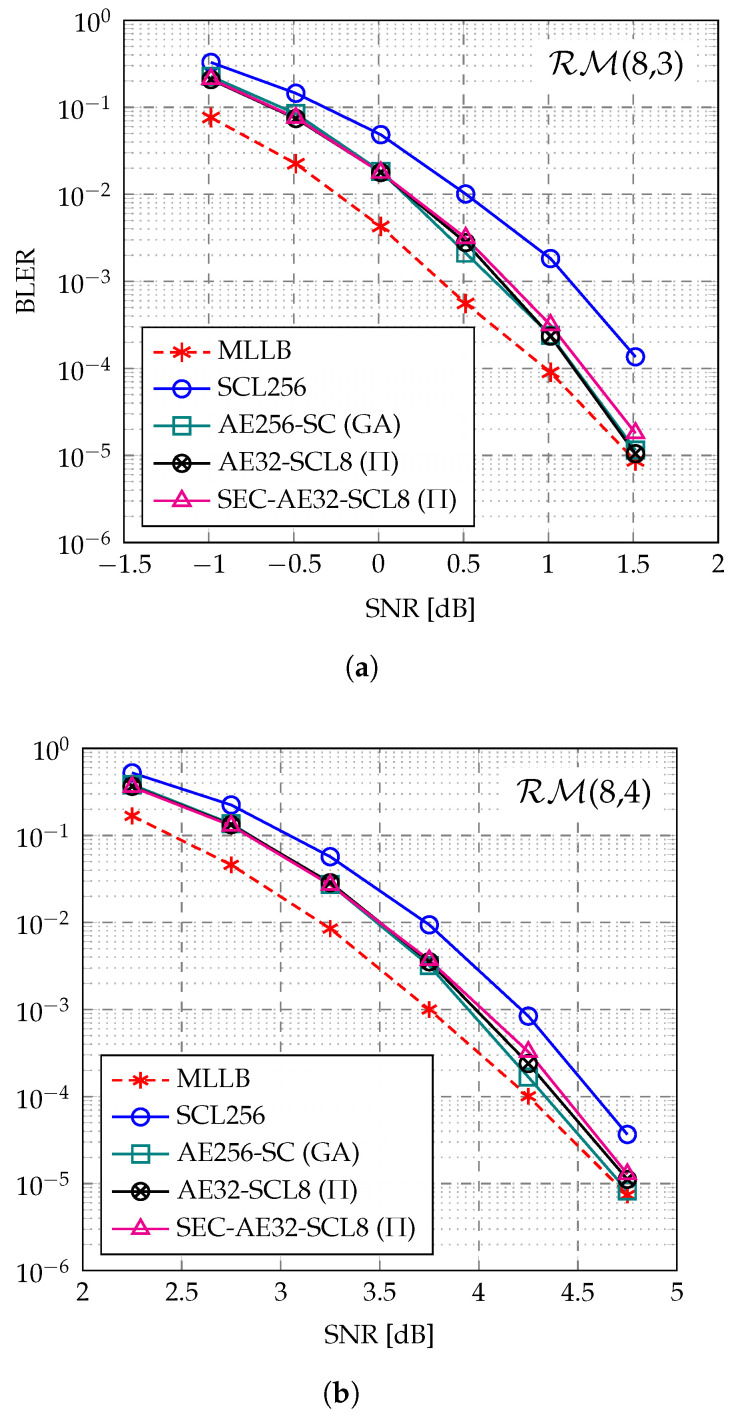
BLER comparison of MLLB [19], SCL256 decoder [32], AE256-SC (GA) decoder [21], AE32-SCL8 (Π) decoder, and the proposed SEC-AE32-SCL8 (Π) decoder for (**a**) RM(8,3) and (**b**) RM(8,4).

Figure 6 portrays the BLER performance of various decoders for the RM(8,3) and RM(8,4) codes. The MLLB is evaluated using the AE256-SC (GA) decoder. As shown in Figure 6a,b, the AE256-SC (GA) decoder outperforms the SCL256 decoder across a wide range of SNR for both RM(8,3) and RM(8,4) codes and approaches the MLLB as the SNR increases. Additionally, the AE32-SCL8 (Π) decoder demonstrates similar BLER performance to the AE256-SC (GA) decoder. By employing the proposed SEC-aided path pruning, the SEC-AE32-SCL8 (Π) decoder exhibits negligible BLER loss compared to the AE32-SCL8-Π decoder, maintaining near ML performance down to a low BLER of around $10^{-5}$.

Figure 5 and Figure 6 both demonstrate that, for short RM codes, the AED is an attractive decoding method. Despite the restriction on the selection of automorphisms in this work, AED can still achieve near ML performance with relatively practical complexity. However, for longer codes, the ensemble size required for AED to achieve near ML performance grows exponentially with the code length, similar to SCL decoding [25]. Even when combined with low-complexity constituent decoders like SC, this still results in high complexity. Therefore, AED is particularly well-suited for short RM codes.

The proposed SEC-aided path pruning technique is primarily designed to reduce the complexity while maintaining the excellent performance of the AED. The SEC-aided path pruning does not significantly impact the AED decoding performance for two main reasons. First, when SEC occurs within some permutation group, it indicates that all constituent decoders in this group output the same decoding result during the halfway decoding process. This can be seen as a redundancy in the decoding capability within the group and suggests that the noise is relatively weak from the observation of this group, as multiple noise realizations do not alter the subcode decoding result. Second, the uniform selection of automorphisms naturally divides the used automorphisms into equal (or nearly equal) groups. If SEC occurs in multiple groups, it means that the error pattern can be decoded to the same partial result under diverse noise transformations. This suggests that the corrupted codeword is decodable in multiple perspectives of observations, which indicates that the decoding of the current codeword decoding does not require a strong diversity gain for error correction. Therefore, when multiple groups exhibit SEC, it can be assumed that only a portion of the automorphisms are needed for correct decoding. In this case, we retain only one constituent decoder from each of the *T* groups that exhibit SEC and prune the remaining M−T constituent decoders. The retained constituent decoders are highly likely to include those that can successfully recover the codeword, thus not significantly impacting the overall performance.

Another perspective on the effectiveness of the proposed SED-aided path pruning is that, when the SNR increases to a certain level, most constituent decoders can recover the transmitted codeword in the majority of cases. When all constituent decoders within the same permutation group correctly decode the codeword, this group will inevitably exhibit SEC during the decoding process. Therefore, the more groups that exhibit SEC at runtime, the higher the possibility that most constituent decoders can correctly decode the codeword. Consequently, the SEC intensity typically reflects the noise level. When the SEC intensity is sufficiently high and exceeds the threshold *T*, a low noise level can be assumed, where retaining only a subset of the constituent decoders will not introduce significant performance degradation.

### 4.2. Complexity

**Figure 7:**
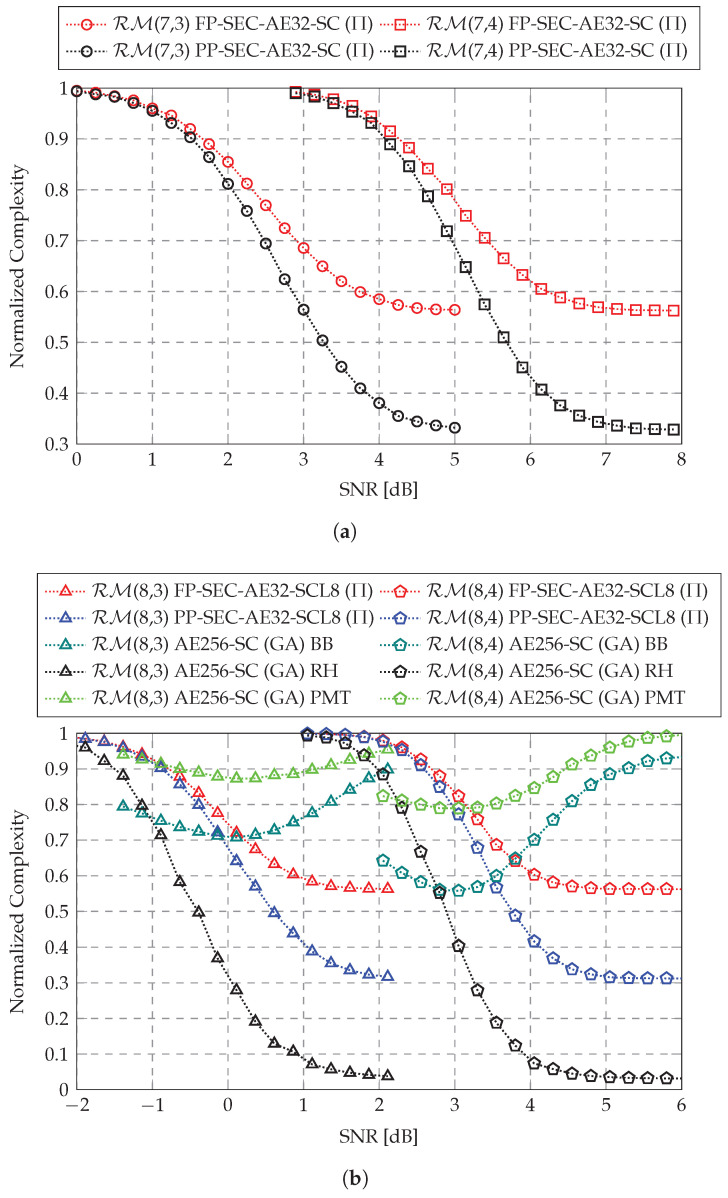
Normalized complexity of (**a**) the proposed SEC-AE32-SC (Π) decoder for the RM(7,3) and RM(7,4) codes and (**b**) the proposed SEC-AE32-SCL8 (Π) decoder and the AE256-SC (GA) decoder using different ET techniques [31] (BB, RH, and PMT) for the RM(8,3) and RM(8,4) codes.

Figure 7 illustrates the variation in the normalized complexity with respect to the SNR in the proposed SEC-AED. As the SNR increases, the complexity under both the FP and PP implementations decreases, approaching their respective lower bounds. Equation (46) indicates that $C_{\mathrm{FP}\text{-}\mathrm{SEC}\text{-}\mathrm{AED}}^{\mathrm{norm},\eta }$ with M=32 has a universal lower bound of 0.5625 for the four considered RM codes, which is validated in Figure 7. However, under the λ configurations used in our experiments, Equation (49) shows that $C_{\mathrm{PP}\text{-}\mathrm{SEC}\text{-}\mathrm{AED}}^{\mathrm{norm},\eta }$ with M=32 has lower bounds of 0.3281 and 0.3125 for the 128-length and 256-length RM codes, respectively. Table 1 summarizes the normalized complexity under the FP and PP schemes at a low BLER of near $10^{-5}$, where the proposed SEC-AED achieves near maximum complexity reduction under the corresponding decoder implementations. Specifically, maximum complexity reductions of 43.5% and 67.6% are observed for the FP and PP implementations, respectively.

In Figure 7b, the normalized complexity of the AE256-SC (GA) decoder for the RM(8,3) and RM(8,4) codes using three different ET techniques is also plotted for comparison. As studied in [31], the repetition detection threshold for the RH method is set to 8, and the path metric threshold for the PMT method is evaluated using a probability of $5\times 10^{-4}$. It can be observed that the BB and PMT methods are effective in the SNR range with medium BLER but do not significantly reduce the complexity in the SNR ranges where BLER is low. Our proposed SEC-AED outperforms the BB and PMT methods in the low BLER SNR region, thereby better adjusting the decoding complexity to adapt to the noise level. The RH method, on the other hand, becomes more effective in reducing complexity as the SNR increases. In the low BLER SNR region, it reduces complexity by up to 96.9% for both the RM(8,3) and RM(8,4) codes. However, the RH method is only feasible under a fully serial AED architecture, which results in much higher worst-case latency compared to the proposed fully parallel and partially parallel SEC-AED. Therefore, our proposed SEC-AED balances decoding latency and complexity, making it more suitable for low-latency scenarios.

### 4.3. Practical Relevance

Notably, the proposed SEC-aided path pruning technique incurs negligible complexity overhead. Firstly, it makes use of the SPCM, which is derived from the LLR-based partial PM of SC and SCL decoding, without the need for additional metric calculations. Secondly, it only depends on a single round of SPCM comparisons among the constituent decoders after half of the bit estimates have been made. Additionally, the SEC-aided path pruning technique is well-compatible with tree pruning techniques that simplify the constituent decoders [26]. This indicates the potential of our proposed approach to be combined with optimized SC or SCL decoding implementations. Through this integration, the AED for RM codes is anticipated to achieve a further reduction in latency and complexity, making it appealing in latency-critical or power-constrained scenarios.

Another advantage of the proposed SEC-aided path pruning technique is its low sensitivity to quantization noise in channel LLRs. This is due to the fact that it relies on the relative characteristics of the SPCMs instead of their absolute values during SC or SCL decoding. Consequently, the SEC-aided path pruning demonstrates excellent stability in quantized hardware implementations of the AED. While we focused on analysis and simulations in this study, future work aims to further validate the practical applicability of our method. In particular, we plan to implement the proposed SEC-AED on application-specific integrated circuit (ASIC) or field-programmable gate array (FPGA) platforms to analyze its power consumption and performance scalability across different RM codes. This endeavor will yield a comprehensive evaluation of the potential of the proposed SEC-AED for real-world applications.

### 4.4. Extensibility

It is well known that AED utilizing the full automorphism group for permutation selection achieves superior performance for RM codes [21]. However, this approach cannot guarantee the PI property in information set partitioning due to complicated affine bijections, making our proposed SEC-aided path pruning method inapplicable. To address this limitation, we may explore a hybrid design that combines both FGPG and non-FGPG automorphisms. By carefully adjusting the proportion between these two types of permutations, this approach aims to simultaneously enhance AED performance while enabling the application of our SEC-aided path pruning pruning method in part of the constituent decoders. The SEC intensity basically functions as an online noise evaluation technique in AED. It enables exploration of new complexity reduction strategies, representing a promising direction for extended research. Additionally, while AED is also applicable to polar codes [36], their automorphisms also lack guaranteed PI properties. Nevertheless, certain specific automorphism structures in polar codes may partially satisfy PI requirements. Through careful analysis of these structures, we can further investigate pruning techniques tailored for the AED of polar codes.

## 5. Conclusions

This paper proposes an improved automorphism ensemble decoder (AED) for RM codes, incorporating a novel subcode estimate convergence (SEC)-aided path pruning technique to reduce complexity. We focus on a specific subgroup of the full automorphism group of RM codes, namely the factor graph permutation group (FGPG), to randomly select permutations for the AED. By partitioning the FGPG into subgroups based on the affine bijection permutation matrices of automorphisms, we prove that the Plotkin construction-based information set partitioning for RM codes exhibits permutation invariance (PI) among the automorphisms from the same subgroup of FGPG. This PI property facilitates the SEC phenomenon among the SC or SCL constituent decoders in AED. Observing that a strong SEC typically indicates a low noise level, where the full decoding capacity of AED is often unnecessary, we introduce runtime path pruning which allows only a few constituent decoders to continue decoding when the intensity of SEC exceeds a preset threshold. The numerical results demonstrate that, at a low BLER of around $10^{-5}$, the proposed SEC-aided path pruning technique achieves complexity reductions of up to 43.5% and 67.6% under fully parallel and partially parallel AED implementations, respectively, while maintaining near ML performance for various short RM codes. The SEC-aided path pruning technique can serve as an efficient power-reduction technology for hardware-based AED implementations, enhancing the potential of RM codes for use in IoT applications that demand high reliability, low latency, and low complexity. Future work aims to further evaluate the practical applicability of our method through hardware validation.

## Figures and Tables

**Table 1:** Normalized complexity comparison between the FP and PP schemes for the proposed SEC-AED (Π) at a BLER of near $10^{-5}$.

Code	*N*	*K*	*R*	*M*	D	SNR [dB]	FP	PP
RM(7,3)	128	64	0.50	32	SC	4.0	58.5%	38.1%
RM(7,4)	128	99	0.77	32	SC	7.2	56.5%	33.6%
RM(8,3)	256	93	0.36	32	SCL8	1.5	56.7%	33.6%
RM(8,4)	256	163	0.64	32	SCL8	4.7	56.5%	32.4%

## Data Availability

The original contributions found in the study are included in the article. Further inquiries can be directed to the corresponding author.
